# Obstetric complications, cortical gyrification, and cognition in first-episode psychosis

**DOI:** 10.1017/S0033291725100974

**Published:** 2025-07-22

**Authors:** Ana Costas-Carrera, Norma Verdolini, Gisela Mezquida, Maria Florencia Forte, Joost Janssen, Clemente Garcia-Rizo, Anabel Martinez-Aran, Pablo Andres-Camazon, Ana Maria Sánchez-Torres, Daniel Berge, Elena de la Serna, Rafael Penades, Isabel Valli, Silvia Amoretti, PEPs Group

**Affiliations:** 1 Hospital Universitario Central de Asturias (HUCA), Oviedo, Spain; 2 Medicine Department, University of Barcelona, Barcelona, Spain; 3 Department of Mental Health, Umbria 1 Mental Health Center, Perugia, Italy; 4 Serra-Hunter Fellow, Department of Basic Clinal Practice, Pharmacology Unit, University of Barcelona, Barcelona, Spain; 5 Barcelona Clínic Schizophrenia Unit (BCSU), Neuroscience Institute, Hospital Clinic de Barcelona, Barcelona, Spain; 6 Biomedical Research Networking Center for Mental Health Network (CIBERSAM), Madrid, Spain; 7 Institut d’Investigacions Biomèdiques August Pi i Sunyer (IDIBAPS), Barcelona, Spain; 8 Bipolar and Depressive Disorders Unit, Hospital Clinic de Barcelona, Institute of Neurosciences, Barcelona, Spain; 9 Department of Child and Adolescent Psychiatry, Institute of Psychiatry and Mental Health, Hospital General Universitario Gregorio Marañón, IiSGM, School of Medicine, Universidad Complutense, Madrid, Spain; 10 Department of Health Sciences, Universidad Pública de Navarra, Pamplona, Spain; 11 Instituto de Investigación Sanitaria de Navarra (IdiSNA), Pamplona, Spain; 12 Institut de Salud Mental, Hospital del Mar, Barcelona, Spain; 13 Hospital del Mar Research Institute, Barcelona, Spain; 14 Department of Medicine and Life Sciences, Universitat Pompeu Fabra (UPF), Barcelona, Spain; 15 Department of Child and Adolescent Psychiatry and Psychology, Institut Clinic de Neurociències, Hospital Clínic Universitari, Barcelona, Spain; 16 Institute of Psychiatry Psychology and Neuroscience, King’s College London, London, UK; 17 FIDMAG, Germanes Hospitalaries Research Foundation, Barcelona, Spain; 18 Group of Psychiatry, Mental Health and Addictions, Vall d’Hebron Research Institute (VHIR), Barcelona, Spain

**Keywords:** cognition, cortical folding, first-episode psychosis, gyrification, magnetic resonance imaging, schizophrenia, obstetric complications, delivery, intrauterine period, epiphenomena, verbal memory, working memory

## Abstract

**Background:**

Obstetric complications (OCs) are associated with cognitive and brain abnormalities observed in patients with schizophrenia. Gyrification, a measure of cortical integrity sensitive to events occurring during the prenatal and perinatal periods, is also altered in first-episode psychosis (FEP). We examined the relationship between OCs and gyrification in FEP, as well as whether gyrification mediates the relationship between OCs and cognition.

**Methods:**

We examined differences in the Local Gyrification Index (LGI) for the frontal, parietal, temporal, occipital, and cingulate cortices between 139 FEP patients and 125 healthy controls (HCs). Regression analyses explored whether OCs and diagnosis interact to explain LGI variation. Parametric mediation analyses were conducted to assess the effect of LGI on the relationship between OCs and cognition for FEP and HC.

**Results:**

Significant LGI differences were observed between FEP patients and HC in the left parietal and bilateral cingulate and occipital cortices. There was a significant interaction between OCs and diagnosis on the left cingulate cortex (LCC) that was specific to males (*p* = 0.04) and was driven by gestational rather than intrauterine OCs.

In HCs, OCs had a direct effect on working memory (WM) (*p* = 0.048) in the mediation analysis, whereas in FEP, we observed no significant effect of OCs on either verbal or WM.

**Conclusions:**

OCs interact with diagnosis to predict LCC gyrification, such that males with FEP exposed to OCs exhibit the lowest LGI. OCs influence WM, and LCC gyrification may mediate this relation only in HC, suggesting a differential neurodevelopmental process in psychosis.

## Introduction

Schizophrenia results from the complex interplay of genetic and environmental factors, which can impact the trajectories of brain development, particularly over the prenatal and perinatal stages, due to the important brain changes that occur during the intrauterine and the early postnatal period (Sasabayashi, Takahashi, Takayanagi, & Suzuki, 2021). Obstetric complications (OCs) have been associated with the neural network abnormalities observed in schizophrenia (Keshavan & Hogarty, 1999). Exposure to OCs has been reported to influence the subsequent development of schizophrenia with an odds ratio between 1.17 and 3.5, and the largest effect sizes observed for polyhydramnios, premature rupture of membranes, low birth weight, and birth hypoxia (Davies et al., 2020).

Reduced gray matter volume is a well-established feature of schizophrenia (De Peri et al., 2012; Ellison-Wright, Glahn, Laird, Thelen, & Bullmore, 2008), especially in regions such as the frontal and temporal cortices, the cingulate, thalamus, and hippocampus (Matsuda & Ohi, 2018). However, measures of cortical morphology, such as surface area (SA), cortical thickness (CTh), and gyrification, exhibit distinct maturational trajectories (Smith et al., 2015). The latter refers to the development of brain surface folding patterns that, based on curvature and sulcal morphology, contribute to cortical SA (Fernández & Borrell, 2023; Tonya White, Su, Schmidt, Kao, & Sapiro, 2010). Gyrification begins between 10 and 15 weeks of gestation and continues through the early postnatal period, remaining relatively stable thereafter (White et al., 2010). In contrast, maturational changes in SA and CTh exhibit a more protracted timeline, continuing throughout adolescence (Zilles, Palomero-Gallagher, & Amunts, 2013). Therefore, abnormalities in gyrification may reflect prenatal or perinatal adverse events (Papini et al., 2020; Smith et al., 2015) and could be used as markers of early neurodevelopment (Nelson et al., 2020).

In healthy controls (HCs), cortical gyrification is related to general cognitive ability (Papini et al., 2020) and with language and working memory (WM) domains (Schmitt et al., 2023). Exposure to obstetric risk factors, such as very preterm birth, impacts the Local Gyrification Index (LGI) and is related to poorer general cognitive ability, with cognitive deficits and higher rates of psychopathology (Papini et al., 2020; Schmitt et al., 2023).

In schizophrenia, abnormal gyrification is considered an endophenotype reflecting aberrant connectivity (White & Gottesman, 2012). Earlier sulci of the brain are thought to be correlated with genetic influences, while tertiary sulci are associated with nongenetic exposures to environmental risk factors (Sasabayashi, Takahashi, Takayanagi, & Suzuki, 2021). Abnormal gyrification has been described both in the at-risk mental state (ARMS) and first-episode psychosis (FEP), suggesting it could be an early imaging biomarker for psychosis (Matsuda & Ohi, 2018; Sasabayashi et al., 2021; White & Gottesman, 2012).

In chronic schizophrenia, gyrification abnormalities were reported to be very heterogeneous (White & Gottesman, 2012). Some studies have reported increased LGI, which refers to the ratio between the complete superficial contour and the outer contour of the cortex (Matsuda & Ohi, 2018) in regions such as the prefrontal cortex, insula, and temporo-parieto-occipital cortices (Sasabayashi et al., 2021; Spalthoff, Gaser, & Nenadić, 2018; White & Gottesman, 2012). In contrast, other studies have found decreased LGI (Matsuda & Ohi, 2018; Nelson et al., 2020; Nesvåg et al., 2014; Palaniyappan & Liddle, 2012; Palaniyappan et al., 2013; Rychagov et al., 2024) predominantly in frontotemporal regions (Nanda et al., 2014; Palaniyappan, Mallikarjun, Joseph, White, & Liddle, 2011; Sasabayashi et al., 2021), including the insula, superior temporal gyrus, Broca’s area, left precentral gyrus, right middle temporal gyrus, precuneus, and cingulate cortex (Madeira et al., 2020; Matsuda & Ohi, 2018; Nanda et al., 2014; Nesvåg et al., 2014; Wheeler & Harper, 2007). Inconsistencies were considered to reflect methodological differences, heterogeneity in demographic and clinical variables, or abnormalities being specific depending on the brain region examined (Si et al., 2024).

Heterogeneity in gyrification abnormalities has also been reported in the early stages of psychosis, with several studies in individuals with FEP and ARMS reporting hypergyria in various brain regions (Narr et al., 2004; Sasabayashi et al., 2021) and also hypogyria in the frontotemporal and insular cortices (Harris et al., 2004; Palaniyappan et al., 2013). Zhou et al. (2021) observed a higher LGI in regions such as the left lateral occipital cortex, and lower LGI in other regions such as the left transverse temporal cortex in drug-naïve patients with FEP (Matsuda & Ohi, 2018; H. Zhou et al., 2021). Sasabayashi et al. reported increased LGI in the left medial occipital cortex of individuals with ARMS who subsequently developed psychosis and considered it as a possible endophenotype of subsequent transition (Sasabayashi et al., 2017).

In schizophrenia, several brain abnormalities have been related to OCs exposure (Costas-Carrera, Garcia-Rizo, Bitanihirwe, & Penadés, 2020), including reduced cortical volume (Cannon et al., 2002; Neilson et al., 2018; Smith et al., 2015), reduced hippocampal volume (McNeil, Cantor-Graae, & Weinberger, 2000; Schulze et al., 2003; Stefanis et al., 1999), and an increased ventricle–brain ratio (Cannon et al., 2002; Costas-Carrera et al., 2024; McNeil et al., 2000; Neilson et al., 2018). Only three studies analyzed the relationship between OCs and gyrification in patients with schizophrenia (Falkai et al., 2007; Haukvik et al., 2012; Smith et al., 2015). Falkai et al. observed no effect of OC exposure on gyrification in schizophrenia when using a two-dimensional method, whereas Haukvik et al. (2012) and Smith et al. (2015) reported a relationship between OCs and hypogyria . However, only Smith et al. (2015) observed that the relationship between OCs and hypogyria was specific to patients with FEP and not in HCs (Smith et al., 2015).

Gyrification has been associated with cognition in both HCs (Gautam, Anstey, Wen, Sachdev, & Cherbuin, 2015; Green et al., 2018; Schmitt et al., 2023) and patients diagnosed with schizophrenia (Sasabayashi et al., 2017). Gautam et al. observed that greater gyrification of the lateral frontal cortex was associated with better performance in terms of executive function in HCs (Gautam et al., 2015), whereas Green et al. observed a positive relationship between measures in the parietal–frontal regions and WM in HCs (Green et al., 2018). In patients with schizophrenia, greater gyrification of the right frontal cortex was associated with worse executive function (Sasabayashi et al., 2021), supporting an aberrant neurodevelopment in schizophrenia. Fetal growth is also correlated with cognition, and variables such as birth weight and gestational age are considered markers of the intrauterine environment (Schmitt et al., 2023). Gestational age moderates the association between gyrification in regions such as the left supramarginal gyrus and the left superior frontal gyrus with attention/WM (Schmitt et al., 2023). In adults born premature, increased frontal–temporal–parietal gyrification was related to worse cognitive performance (Hedderich et al., 2019). Papini et al. reported widespread hypogyria in adults born preterm, especially in the middle and inferior frontal, superior temporal, and medial occipito-parietal regions, and found a relationship between LGI and cognition, with the spatial distribution of these associations substantially differing between preterm-born individuals and HCs (Papini et al., 2020).

OCs exert a differential impact on fetal outcomes regarding sex, suggesting a sexual dimorphism effect, which implies different outcomes regarding the timeframe of the event (Yu, Chen, Ge, & Wang, 2021). This effect is particularly relevant in schizophrenia, as sex-related brain structural and cognitive differences have been described (Mendrek & Mancini-Marïe, 2016).

The assessment of FEP patients provides valuable insights into how adverse prenatal and perinatal factors may contribute to neurodevelopmental vulnerabilities that increase the risk of psychosis without the potential confounding effect of protracted illness and medication exposure. We, therefore, sought to examine the impact of OCs on gyrification in FEP patients and their relationship with cognitive performance.

We hypothesized that there would be differences in gyrification between FEP patients and HCs, with a lower LGI in FEP patients, and that these differences would be related to OC exposure. We also hypothesized that cortical folding patterns would play a role in the relationship between OCs and cognition, and examined the potential effect of sex and the timeframe of OC exposure.

## Methods and materials

This research was conducted as part of a multicenter longitudinal study examining gene–environment interactions on the pathway to psychosis (the PEPs study, ‘Phenotype–genotype and environmental interaction: Application of a predictive model in first psychotic episodes’, Bernardo et al. (2013)).

### Participants

The sample of the PEPs study included 335 FEP patients and 253 HCs recruited between January 2009 and December 2011. The inclusion criteria and characteristics of the study have been previously described in detail (Salagre et al., 2019). Briefly, subjects with FEP aged 7–35 years, presenting psychotic symptoms for <12 months, were recruited from the inpatient and outpatient units of 16 participating Spanish centers, 14 of which are members of the Center of Biomedical Research Network on Mental Health (CIBERSAM). HCs were recruited at each site through advertisements and matched with patients by age (within ±10%), sex, and parental socioeconomic status, as measured by the Hollingshead–Redlich scale (within ±1 level). For the neuroimaging component of the study, a maximum of 6 months was established from inclusion to scan time. All centers received the approval of their respective Independent Ethics Committee. All procedures contributing to this work comply with the ethical standards of the relevant national and institutional committees on human experimentation and with the Declaration of Helsinki of 1975, as revised in 2008. Written informed consent was obtained from all participants before they participated in the study and from parents/legal guardians for children under 16 years of age (children gave assent). In the present study, from the total sample, we included 139 individuals with FEP and 125 HCs based on the availability of data for magnetic resonance imaging, cognitive assessments, and OC exposure.

### History of OC assessment

OCs were assessed using the Lewis–Murray scale through a family interview (Lewis & Murray, 1987). The scale groups OCs into three categories, A, B, and C, according to the type of complication defined as follows (M. Cannon, Jones, & Murray, 2002; Mezquida et al., 2018):

- Complications of pregnancy: syphilis or rubella, rhesus isoimmunization/Rh incompatibility, severe preeclampsia requiring hospitalization or induction of labor, and bleeding before delivery or threatened abortion.
- Abnormal fetal growth and development: twin delivery, preterm birth before 37 weeks, or long-term after 42 weeks, weight at birth <2500 g, and any important physical abnormality.
- Difficulties in delivery: premature rupture of membranes, duration of delivery more than 36 h or less than 3 h, umbilical cord prolapse, complicated cesarean delivery, abnormal fetal presentation, use of forceps, and being in an incubator for more than 4 weeks.

Participants were stratified based on exposure, which the Lewis–Murray scale classifies into definite and dubious based on the quality of the information. We included only definite events (yes/no) grouped as intrauterine (Lewis A and B) and delivery complications (Lewis C), as well as the total score (Lewis T).

### Image acquisition and processing

After all DICOM images were converted to NIFTI format using the dcm2niix function from MRIcron software, the FreeSurfer analysis package (v7.1.1, https://surfer.nmr.mgh.harvard.edu/) was used to generate measurements of CTh, cortical and subcortical volumes, and LGI. The standard FreeSurfer processing pipeline was employed, which follows the workflow: motion and bias field correction, skull extraction, affine and nonlinear alignment to the Talairach atlas, subcortical division, and cortical segmentation using the Desikan–Killiany atlas. For quality assurance, a visual inspection of the segmentation was performed by a technician specialized in neuroimaging, following the quality control protocol 2.0 of the ENIGMA consortium (https://enigma.ini.usc.edu/protocols/imaging-protocols).

The LGI was calculated using FreeSurfer with default parameters (vertex-wise values were averaged for each lobe [frontal, parietal, temporal, and occipital] and area [cingulate cortex], considering the right and left hemispheres separately, Kernel size 10 mm; Madan & Kensinger (2017)).

In this multicenter study, data were collected from six distinct neuroimaging centers using different scanners (Siemens Magnetom Trio Tim 3T, Siemens Symphony 1.5T, Philips Achieva 3T, Philips Intera 1.5T, GE Signa Horizon MX 1.5T, and GE Signa Excite 1.5T). Detailed information on the acquisition parameters from each participating platform can be found in previous work from the collaborative study (Pina-Camacho et al., 2016). To adjust for site, we employed the ComBat batch harmonization method (Fortin et al., 2018).

### Cognitive assessment

In the PEPs study, the cognitive assessment at baseline was performed in the second month after inclusion to ensure the clinical stability of patients after the FEP (Bernardo et al., 2013; Cuesta et al., 2015). For our hypothesis, we focused on verbal memory (VM) and WM, two cognitive domains that we had previously observed to be associated with OC exposure in schizophrenia (Amoretti, Rabelo-da-Ponte, et al., 2022).

VM was assessed using the Verbal Learning Test España-Complutense (Benedet, 1998), while WM was evaluated using the Digit Span and Letter-Number Sequencing subtests of the WAIS-III (Wechsler, 2019). These cognitive domains were derived from our previous work using principal component analysis (Amoretti, Rosa, et al., 2022), with higher scores reflecting better performance in both domains. Further details are described in a previous work (Cuesta et al., 2015).

### Statistical analysis

Descriptive statistics were calculated for each sociodemographic, neuropsychological, and clinical variable. Continuous variables are presented as mean value ± standard deviation and compared using Student’s *t*-tests. Categorical variables are expressed as total numbers (or percentages) and compared between groups using *χ*^2^-tests. OCs were reported as dichotomous variables (yes/no).

Student’s *t*-tests were used to examine differences in LGI between FEP patients and HCs. General linear model (GLM) analyses were performed to test the relationship between each independent variable and the LGI of each brain area. Independent variables were sex, age, and chlorpromazine equivalent antipsychotic dose (Gardner, Murphy, O’Donnell, Centorrino, & Baldessarini, 2010), diagnostic group (FEP/HCs), OCs (presence/absence), and the interaction between OCs and diagnostic group. We report results before and after false discovery rate (FDR) correction for multiple comparisons.

For cognitive measures, parametric mediation analyses were conducted in both groups (HCs and patients with FEP) to test whether gyrification mediates the effect of OCs on cognitive outcomes. We employed the LGI of areas where we had observed a significant interaction between diagnosis and OCs as the mediation variable between the independent variable (OCs) and cognitive outcomes, including WM and VM. The estimation of average causal mediation effects, average direct effects, total effects, and proportion-mediated effects was computed for each of the 1,000 bootstrapped resamples, and the 95% confidence interval (95% CI) was computed by determining the 2.5th and 97.5th percentiles for the resamples. The mediation analysis was performed with the mediation package version 4.5.03 (Tingley, Yamamoto, Hirose, Keele, & Imai, 2014).

Statistical analyses were performed with Statistical Package for the Social Sciences (Version 25) and R version 4.5.0.

## Results

### Sociodemographic and clinical variables

The sociodemographic characteristics of the sample are described in Table 1. There were no significant between-group differences in sex or age. However, as expected, the groups differed significantly in terms of educational level (*χ*^2^ 33.32, *p* < 0.001), with higher levels in HCs than in patients. There was a trend toward significance in the prevalence of OCs (Lewis total score) (*χ*^2^ = 2.91, *p* = 0.089) between FEP and HCs. The median duration of untreated psychosis for the FEP patients was 45 days, and the daily equivalent dose of chlorpromazine was 450 (mean value: 557.62).

**Table 1.** Clinical and sociodemographic characteristics of the sample

	First-episode psychosis (*n* = 139)	Healthy controls (*N* = 125)	*χ*^2^	p
**Categorial variables**				
Sex - Female - Male	45 (32%) 94 (68%)	47 (38%) 78 (62%)	0.79	0.374
Educational level - Basic education - Secondary education - Graduate/postgraduate education	34 (24%) 82 (59%) 23 (17%)	11 (9%) 56 (45%) 58 (46%)	33.32	<0.001
Lewis–Murray total - Yes - No	34 (25%) 105 (75%)	20 (16%) 105 (84%)	2.91	0.089
Lewis–Murray A - Yes - No	7 (5%) 129 (95%)	2 (2%) 121 (98%)	2.39	0.122
Lewis–Murray B - Yes - No	9 (7%) 129 (94%)	9 (7%) 116 (93%)	0.05	0.828
Lewis–Murray C - Yes - No	24 (18%) 111 (82%)	14 (11%) 110 (89%)	2.17	0.140

	(Mean ± SD)	(Mean ± SD)	T	*p*
**Continuum variables**				
Age (years)	23.61 ± 6.04	23.95 ± 6.12	−0.45	0.325
Verbal memory	193.92 ± 74.47	281.52 ± 44.05	−11.11	<0.001
Working memory	70.92 ± 15.13	85.77 ± 15.21	−7.65	<0.001
CPZ equivalents	557.62 ± 417.13			
Left occipital LGI	2.80 ± 0.12	2.83 ± 0.16	−1.93	0.06
Right occipital LGI	2.87 ± 0.13	2.90 ± 0.17	−1.66	0.10
Left frontal LGI	2.88 ± 0.14	2.92 ± 0.15	−1.41	0.04
Right frontal LGI	2.88 ± 0.14	2.92 ± 0.15	−1.10	0.13
Left parietal LGI	3.30 ± 0.15	3.34 ± 0.18	−1.18	0.06
Right parietal LGI	3.31 ± 0.16	3.35 ± 0.19	−0.85	0.10
Left temporal LGI	3.34 ± 0.17	3.37 ± 0.18	−1.16	0.12
Right temporal LGI	3.27 ± 0.16	3.32 ± 0.18	−1.68	0.03
Left cingulate LGI	2.32 ± 0.11	2.34 ± 0.17	−1.16	0.12
Right cingulate LGI	2.35 ± 0.12	2.39 ± 0.17	−1.45	0.09

Lewis–Murray total score (any difficulty during delivery and pregnancy).

Lewis–Murray A: Complications of pregnancy; Lewis–Murray B: Abnormal fetal growth and development; Lewis–Murray C: Difficulties in delivery.

### Differences in LGI between FEP and HC

We observed significant differences in LGI between FEP patients and HCs in the left cingulate (Wald’s *χ*^2^ = 4.24, *p* = 0.04), left occipital (Wald’s *χ*^2^ = 6.47, *p* = 0.01), left parietal (Wald’s *χ*^2^ = 3.93, *p* = 0.05), right cingulate (Wald’s *χ*^2^ = 3.77, *p* = 0.05), and right occipital (Wald’s *χ*^2^ = 5.95, *p* = 0.01) cortices (Table 2).

**Table 2.** Generalized linear model gyrification index lobes by diagnosis (psychosis/control) and stratified by the presence/absence of difficulties during the intrauterine/delivery period (OC presence/absence)

	Diagnosis				Effect diagnosis		Effect OCs		Effect diagnosis by OCs		
	First episode psychosis		Healthy controls								
	OCs−	OCs+	OCs−	OCs+	Wald’s *χ*^2^	*P*-value	Wald’s *χ*^2^	*P*-value	Wald’s *χ*^2^	*P*-value	FDR-adjusted
Left frontal LGI	2.87 ± 0.01	2.87 ± 0.02	2.89 ± 0.01	2.94 ± 0.03	3.48	0.06^	0.94	0.33	1.96	0.16	0.32
Right frontal LGI	2.87 ± 0.01	2.86 ± 0.02	2.89 ± 0.01	2.93 ± 0.03	2.41	0.12	0.07	0.78	1.46	0.23	0.32
Left parietal LGLI	3.29 ± 0.02	3.25 ± 0.02	3.32 ± 0.02	3.34 ± 0.03	3.93	0.05^	0.52	0.47	1.64	0.20	0.32
Right parietal LGI	3.30 ± 0.01	3.25 ± 0.03	3.32 ± 0.02	3.35 ± 0.03	3.28	0.07^	0.53	0.47	2.98	0.08^	0.32
Left temporal LGI	3.32 ± 0.01	3.30 ± 0.03	3.36 ± 0.01	3.36 ± 0.03	2.55	0.11	0.32	0.57	0.39	0.53	0.59
Right temporal LGI	3.26 ± 0.02	3.22 ± 0.03	3.29 ± 0.02	3.33 ± 0.03	2.87	0.09^	0.33	0.56	2.66	0.10	0.32
Left occipital LGI	2.78 ± 0.01	2.77 ± 0.02	2.82 ± 0.01	2.83 ± 0.03	6.47	0.01*	0.17	0.68	0.18	0.66	0.66
Right occipital LGI	2.85 ± 0.02	2.82 ± 0.02	2.89 ± 0.02	2.91 ± 0.03	5.95	0.01*	0.3	0.58	1.04	0.31	0.39
Left cingulate LGI	2.30 ± 0.01	2.28 ± 0.02	2.32 ± 0.01	2.41 ± 0.03	4.24	0.04*	0.85	0.36	6.65	0.01*	0.10
Right cingulate LGI	2.34 ± 0.01	2.34 ± 0.02	2.37 ± 0.01	2.42 ± 0.03	3.77	0.05^	0.72	0.39	1.74	0.19	0.32

FDR, false discovery rate; OCs, obstetric complications (during the intrauterine period and delivery; LGI, Local Gyrification Index.

The effect of the estimates (Wald’s *χ*^2^ and *p*-value) for diagnosis and OCs refers to the outcome of the regression analysis without interaction.

The effect of estimates (Wald’s *χ*^2^ and *p*-value) for diagnosis by OCs refers to the outcome of the regression analysis where the interaction was included in addition to the other independent variables. Estimated marginal means of predicted gyrification index values are adjusted by covariates, age, sex, and chlorpromazine equivalent mean dose.

*p<.05; ^*p*=0.05-.10

### Differences in LGI between subjects with and without OCs

Within the whole sample, there were significant differences between subjects with and without OCs in the left frontal (*t* = −2.18, *p* = 0.03); left cingulate (*t* = −2.03, *p* = 0.04); and trend level differences in the right cingulate (*t* = −1.9, *p* = 0.06) cortices (data not shown). However, when we adjusted for covariates (age, sex, and chlorpromazine equivalent mean dose), none of these differences maintained statistical significance (Table 2).

### Association between LGI, diagnosis, and OCs

A GLM was applied to analyze whether OC exposure (Lewis total score) interacted with diagnosis to predict differences in LGI. We observed a significant interaction between OCs and diagnosis in the left cingulate cortex (LCC) (Wald’s *χ*^2^ = 6.65, *p* = 0.01), and trend level differences in the right parietal cortex (Wald’s *χ*^2^ = 2.98, *p* = 0.08). However, there was no significant effect on LGI after adjusting for multiple comparisons.

**Figure 1.**
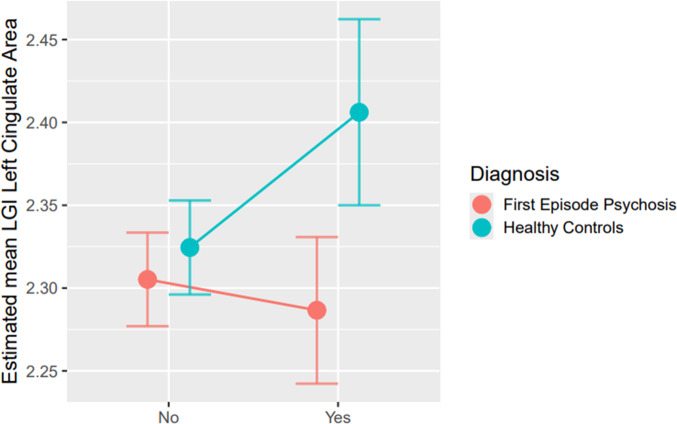
General linear model mean predicted estimated value for Local Gyrification Index for the left cingulate area covariated by sex, age, chlorpromazine equivalent dose, diagnostic group (FEP patients/HCs), OCs (presence/absence), and the interaction between OCs and the diagnostic group. *Note*: FEP, first episode psychosis; HC, healthy controls; OCs, obstetric complications.

In the LCC, FEP patients with OCs displayed the lowest LGI (2.28 ± 0.02), followed by FEP patients without OCs (2.30 ± 0.01), HCs without OCs (2.32 ± 0.01), and finally, HCs with OCs (2.41 ± 0.03) (Table 2 and Figure 1).

We then separately analyzed the role of gestational (Lewis A + B) and delivery complications (Lewis C) on LGI and observed that the interaction we had found in LCC was specific to those participants who had suffered complications during gestation (Wald’s *χ*^2^ = 5.31, *p* = 0.02) rather than delivery (Wald’s *χ*^2^ = 3.231, *p* = 0.072).

Finally, we examined whether sex influenced this interaction effect on LGI in the LCC. In the MLG model, sex had a significant effect on LGI (Wald’s *χ*^2^ = 14.89, *p* < 0.001) and the interaction of Lewis T × diagnosis × sex was significant (Wald’s *χ*^2^ = 9.70, *p* = 0.021). Therefore, we stratified the sample according to sex and observed that the effect of Lewis T significantly interacted with diagnosis only in males (Wald’s *χ*^2^ = 8.12, *p* = 0.004) and remained significant after FDR correction (*p* = 0.04) (Supplementary Table 2A). In this male subsample, FEP with OCs displayed the lowest LGI (2.33 ± 0.03), followed by FEP without OCs (2.31 ± 0.03), HCs without OCs (2.36 ± 0.02), and finally, HCs with OCs (2.50 ± 0.04). This effect was not observed in the female group (Wald’s *χ*^2^ = 0.253; *p* = 0.615) (Supplementary Table 2B). However, the female sex was underrepresented, with only 17 women with OC history.

### Association between gyrification, OCs, and cognition

To analyze the relationship between OCs, cognition, and gyrification, we focused on the LCC, where we observed a significant interaction between diagnosis and OCs.

We tested a mediation model, with OCs as the independent variable, cognitive functioning (independently examining VM and WM) as the dependent variable, and the LGI in the LCC as the mediator. We stratified the sample between patients and HCs, as previous studies have shown different profiles.

In HCs (Supplementary Figure S2), the total effect of OCs (Lewis T) on WM was not significant (*β* = −5.46, 95% CI: −13.45 to 1.86; *p* = 0.14), nor was the average causal mediation effect (ACME) (*β* = 1.68, 95% CI: −0.11 to 4.32; *p* = 0.10), whereas the average direct impact of OCs on WM (ADE) (*β* = −7.14, 95% CI: −15.16 to −0.26; *p* = 0.048*) was significant. On the contrary, no significant effects were found for VM: total effect (*β* = 10.92, 95% CI: −10.94 to 28.81; *p* = 0.32), ACME (*β* = −1.79, 95% CI: −6.20 to 2.40; *p* = 0.38), or ADE (*β* = 12.71, 95% CI: −9.26 to 30.41; *p* = 0.29).

In FEP patients (Supplementary Figure 3), none of the effects were significant for WM: total effect (*β* = −0.66, 95% CI: −7.26 to 6.54; *p* = 0.88), ACME (*β* = −0.18, 95% CI: −1.32 to 0.41; *p* = 0.63), and ADE (*β* = −0.48, 95% CI: −7.11 to 6.77; *p* = 0.92). Similarly, no significant effects were found for VM: total effect (*β* = −10.92, 95% CI: −40.16 to 16.41; *p* = 0.40), ACME (*β* = −0.27, 95% CI: −5.80 to 4.31; *p* = 0.92), or ADE (*β* = −10.65, 95% CI: −39.99 to 16.98; *p* = 0.41).

When mediation analyses were stratified by sex (Supplementary Figures 4 and 5), none of the effects were significant for WM in HC males: total effect (*β* = −2.83, 95% CI: −15.49 to 6.84; *p* = 0.66), ACME (*β* = 2.75, 95% CI: −0.28 to 8.41; *p* = 0.10), and ADE (*β* = −5.58, 95% CI: −19.25 to 4.48; *p* = 0.38), while in HC females, a trend was seen in the total effect (*β* = −7.36, 95% CI: −15.35 to 0.54; *p* = 0.07). However, the ACME (*β* = −0.28, 95% CI: −2.69 to 1.44; *p* = 0.75) and ADE (*β* = −7-08, 95% CI: −15.31 to 1.24; *p* = 0.11) in HC females were not significant.

In FEP patients, none of the effects were significant for WM in males: ACME (*β* = −0.11, 95% CI: −1.10 to 0.80; *p* = 0.81), ADE (*β* = 4.15, 95% CI: −3.34 to 12.03; *p* = 0.32), and total effect (*β* = 4.04, 95% CI: −3.47 to 11.85; *p* = 0.33). On the other hand, in female FEP patients, the total effect (*β* = −12.30, 95% CI: −21.57 to −2.15; *p* = 0.03) and ADE (*β* = −11.45, 95% CI: −19.92 to −2.15; *p* = 0.03) were significant, while the ACME (*β* = −0.85, 95% CI: −4.95 to −3.32; *p* = 0.62) was not significant.

## Discussion

This study examined the relationship between exposure to OCs, cortical folding, and cognitive function.

We observed significant differences in LGI measures between FEP and HC participants in the left parietal, bilateral cingulate, and bilateral occipital cortices. These results are consistent with studies that reported hypogyria in patients with psychotic disorders compared to controls (Nanda et al., 2014; Nesvåg et al., 2014), also in the early stages of the illness (Palaniyappan et al., 2013).

In terms of exposure to OCs, regardless of diagnosis, we observed no significant gyrification differences between subjects exposed to OCs and those who were not exposed.

Results of previous studies are inconsistent, with some reporting a relationship between exposure to OCs and a reduction in gyrification (Engelhardt et al., 2015; Haukvik et al., 2012; Smith et al., 2015; Yehuda et al., 2024) and others describing increased gyrification (Hedderich et al., 2019; Wu, De Asis-Cruz, & Limperopoulos, 2024). Such differences may be related to the heterogeneity of OCs (hypoxia, premature birth, fetal growth restriction, etc.).

Therefore, we examined whether prenatal and perinatal complications interacted differently with diagnosis to determine differences in LGI. We observed that a significant interaction with diagnosis to determine LGI differences in the LCC specifically pertained to antepartum complications. This result did not survive correction for multiple comparisons within the entire group but it maintained significance within the male subsample when we stratified our participants based on sex. In this region, male patients exposed to OCs displayed the lowest LGI, while in male HC participants, OCs acted inversely, and exposure was associated with the highest LGI. This finding may reflect how early environmental stressors affect patients and controls differently, suggesting that mere exposure to OCs may not be sufficient to explain the changes observed in psychosis, in keeping with the gene–environment interaction hypothesis. Previous research also reported that exposure to OCs can affect patients with schizophrenia differently compared to HC and specifically identified the smallest hippocampal volumes in patients with OCs (Stefanis et al., 1999). Similarly, Cannon et al. observed that OCs interacted with schizophrenia diagnosis to determine brain volumetric abnormalities such as ventricular enlargement (Cannon et al., 2002).

Ducsay et al. (2018) suggested that hypoxia, a common mechanism of most OCs, is implicated in the disruption of neural plasticity through epigenomic modifications. These alterations in the epigenetic landscape are believed to underlie phenotypic programming mechanisms that influence long-term health outcomes and disease susceptibility (Ducsay et al., 2018). Furthermore, evidence from genome-wide association studies indicates that numerous genes implicated in schizophrenia risk are not only active during fetal neurodevelopment (Hall & Bray, 2022) but are also regulated by hypoxic conditions (Semenza, 2001). In our sample, we observed a specific effect of antepartum complications, which we had previously observed to be significantly associated with case–control status (Valli et al., 2023) and are often characterized by protracted hypoxia-associated placental pathology (Valli & McGuire, 2023).

Collectively, these findings support the hypothesis that exposure to OCs may induce maladaptive epigenetic changes in individuals with a genetic predisposition, thereby altering neurodevelopmental trajectories and heightening vulnerability to subsequent environmental risk factors. On the contrary, the increased LGI in the LCC that we observed in HC exposed to OCs might represent an adaptive mechanism afforded to those at low genetic risk.

Furthermore, within our sample, male participants appeared to be more susceptible to the effects of OCs, as evidenced by a more pronounced reduction in LGI of the LCC in male FEP patients compared to other groups. This finding is consistent with previous studies demonstrating sex-specific differences in brain gyrification patterns in schizophrenia. For instance, one study reported differential gyrification alterations based on sex (Mancini-Marïe et al., 2018), while another found that hypogyrification occurs more frequently in males with schizophrenia than in females (Vogeley et al., 2000). In addition, studies focusing on individuals exposed to OCs have also identified sex-dependent effects, with male patients exhibiting greater LGI reductions across several brain regions (Mareckova, Miles, Andryskova, Brazdil, & Nikolova, 2020; Papini et al., 2020). Overall, males seem to be more vulnerable to intrauterine insults, as also suggested by the differential placental upregulation of genes involved in schizophrenia between males and females exposed to obstetric risk (Sutherland & Brunwasser, 2018; Ursini et al., 2018).

These findings emphasize the importance of considering sex-specific neurodevelopmental trajectories in understanding the impact of OCs on cortical morphology in schizophrenia.

The left cingulate area is involved in affect, attention, memory, and executive function (Haznedar et al., 2004) and is often reported to be altered in schizophrenia (Bersani et al., 2014; Feng et al., 2023). In this region, we observed that FEP with OCs displayed the lowest LGI. The neurodevelopmental model of psychosis (Murray & Lewis, 1987) postulates that genetic predisposition combined with environmental insults may be involved in the aberrant brain development observed in psychosis. In utero insults were identified as key disruptors of neurodevelopmental processes (Keshavan & Hogarty, 1999). In particular, cingulate dysfunction has been suggested to disrupt the modulation of prefrontal–temporal integration (Dazzan et al., 2021; Nanda et al., 2014) with an effect on cognitive functions such as memory and executive function (Fletcher, McKenna, Friston, Frith, & Dolan, 1999; L. Zhou et al., 2016), and a relationship to poorer WM in individuals with FEP (Feng et al., 2023; L. Zhou et al., 2016).

In our sample, FEP patients performed significantly worse than HC participants in both the cognitive domains examined (WM and VM). This is consistent with previous studies reporting these deficits from the early phases of the disorder (Catalan et al., 2024; Rodriguez-Jimenez et al., 2019).

Finally, we examined whether the LGI of the LCC mediated the relationship between OCs and cognitive functioning. In our FEP sample, we did not observe a significant mediation effect for either WM or VM. Meanwhile, a significant direct effect of OCs on WM was observed in HCs. Similarly, the mediation effect of the LCC LGI showed a positive indirect effect on cognitive performance. Although it could be considered a trend toward significance, there is no strong evidence that LCC LGI explains the relationship between OCs and WM. A possible explanation is that cognitive performance in FEP patients depends on a more complex interaction between genetic and environmental risk factors, with several environmental exposures involved in shaping the relationship between OCs and cognition. For instance, a family history of psychosis confers a higher risk for developing psychosis than OCs (Davies et al., 2020) and can contribute to brain changes observed in patients with psychosis or even high-risk populations (Neilson et al., 2018). In addition, other environmental risk factors, such as childhood maltreatment (Sideli et al., 2022) and cannabis use (Yucel et al., 2012), are related to cognitive impairment in FEP patients and may attenuate the isolated effect of OC exposure on cognition.

When stratifying our sample by sex, we observed no significant effect of OCs in relation to cognition in males, while in females, a direct effect of OCs on WM was observed in FEP patients. These differential effects of OCs on cognition by sex were unexpected, especially considering that previous evidence suggests that the male fetus may be more vulnerable to the effects of OCs than the female fetus, and that males who go on to develop psychosis may possess fewer compensatory resources compared to other groups (DiPietro & Voegtline, 2017; Sutherland & Brunwasser, 2018). One possible explanation for this unexpected pattern lies in the underrepresentation of women with a history of OCs within our sample. This limitation increases the likelihood of unstable parameter estimates, inflated effect sizes, and spurious significance, especially in stratified models. Thus, this effect should be interpreted with caution, and future studies with larger and sex-balanced samples are necessary to clarify the role of sex in moderating the relationship between early adversity and cognition in psychosis.

This research should be interpreted in the context of several other limitations. First, OCs have a small effect size to explain psychosis, so larger sample sizes are needed to increase the statistical power and detect significant effects (De Prisco & Vieta, 2024). In addition, this was a cross-sectional study conducted at the onset of psychosis (despite patients being minimally treated, they were not antipsychotic-naïve), so we were not able to control other factors, including treatment adherence (Vieta & De Prisco, 2024). Longitudinal studies of the population could track the long-term impact of OCs on brain and cognitive development, enabling measures to prevent and reduce the risk of developing psychosis and cognitive impairment. In our mediation analysis, we did not include potential confounders, such as educational level or chlorpromazine equivalents (which might be associated with both LGI and cognitive functioning) (Ilzarbe & Vieta, 2023) to reduce the complexity and avoid multicollinearity in the specific case of educational level.

Several strengths counterbalance the aforementioned limitations, such as a relatively large sample size and the lack of confounders associated with the protracted duration of illness and treatment exposure. To the best of the author’s knowledge, this is the first study to examine the potential impact of OCs on brain gyrification processes and how they can affect cognitive performance in FEP.

In summary, we observed that FEP patients differ from HCs in terms of gyrification in the parietal, occipital, and cingulate cortices. Our findings suggest that OCs are associated with altered cortical gyrification, particularly in the LCC, with a differential effect of OC exposure between patients and HCs, especially evident in males and driven by antepartum complications.

Among the HCs, OCs were negatively associated with WM. This pattern potentially reflects compensatory neurodevelopmental mechanisms in response to early adversity, which is also supported by the differential effects of OCs on the brain. In contrast, neither direct nor mediated effects of OCs on cognition were observed among individuals with FEP, suggesting a more complex interplay between early-life adversity, cortical neurodevelopment, and cognition in psychosis. Sex differences may modulate this complex interplay, and the underrepresentation of female participants in our sample limits the strength of our conclusions. Future studies with larger, sex-balanced samples are essential to clarify the role of sex in shaping the neurodevelopmental impact of OCs in psychosis.

## Supporting information

Costas-Carrera et al. supplementary material Costas-Carrera et al. supplementary material

## Supplementary material

The supplementary material for this article can be found at http://doi.org/10.1017/S0033291725100974.

## PEPs Group

Nora Guasch-Capella^5,6^, Anaid Perez-Ramos^5,6,7,19^, Jessica Merchán-Naranjo^6,9^, Laura Pina-Camacho^6,9^, Alexandra Roldán^6,20^, Anna Alonso-Solís^6,20^, Ana González-Pinto^6,21^, Iñaki Zorrilla^6,21^, Pedro M. Ruiz-Lázaro^6,22^, David Vaquero-Puyuelo^6,22^, Maria José Escarti^6,23,24,25^, Marta Perez-Rando^6,24,26^, Anna Mané^6,12,13,14^, Angeles Malagon^6,12,13,14^, Maria Serra-Navarro^7,8^, Eduard Vieta^2,6,7,8^, Josefina Castro-Fornieles^2,6,7,15^, Inmaculada Baeza^2,6,7,15^, Francesco Dal Santo^6,27^, Paz García-Portilla^6,27,28^, Rafael Segarra Echevarria^6,29,30^, Arantzazu Zabala Rabadán^6,29,30^, Roberto Rodriguez-Jimenez^6,31,32^, Isabel Martínez-Gras^31,32,33^, Judith Usall^34^, Anna Butjosa^6,34^, Edith Pomarol-Clotet^6,17^, Raymond Salvador^6,17^, Angela Ibañez^6,35^, Manuel J. Cuesta^10,11^, Vicent Balanzá-Martínez^36^

^19^Neuropsychopharmacology and Psychobiology Research Group, Department of Neuroscience, University of Cadiz, Cadiz, Spain

^20^Department of Psychiatry, Institut d’Investigació Biomèdica Sant Pau, Hospital de la Santa Creu i Sant Pau, Barcelona, Spain

^21^Department of Psychiatry, Hospital Universitario de Alava, UPV/EHU, BIOARABA, Vitoria, Spain

^22^Department of Medicine and Psychiatry, Universidad de Zaragoza, Instituto de Investigación Sanitaria Aragón (IIS Aragón), Zaragoza, Spain

^23^Servicio de Psiquiatria, Hospital Clínico Universitario de Valencia, Valencia, Spain

^24^Fundación Investigación Hospital Clínico de Valencia, INCLIVA, Valencia, Spain

^25^Department of Medicine, University CEU-UCH, Valencia, Spain

^26^Neurobiology Unit, Institute for Biotechnology and Biomedicine (BIOTECMED), Universitat de València, Valencia, Spain

^27^Servicio de Salud del Principado de Asturias (SESPA), Instituto de Investigación Sanitaria del Prinicipado de Aturias (ISPA), Oviedo, Spain

^28^Área de Psiquiatría, Departamento de Medicina, Universidad de Oviedo, Oviedo, Spain

^29^Área de Psiquiatría, Departamento de Neurociencias, Universidad del País Vasco UPV/EHU, Bilbao, Spain

^30^Instituto de Investigación Sanitaria BioCruces Bizkaia, Barakaldo, Spain

^31^Faculty of Medicine, Complutense University of Madrid (UCM), Madrid, Spain

^32^Department of Psychiatry, Instituto de Investigación Sanitaria Hospital 12 de Octubre (imas12), Madrid, Spain

^33^RETIC (Network of Addictive Conditions), Institute of Health Carlos III, Madrid, Spain

^34^Institut de Recerca Sant Joan de Déu, Department of Child and Adolescent Mental Health, Parc Sanitari Sant Joan de Déu, Sant Boi de Llobregat, Spain

^35^Department of Psychiatry, Hospital Universitario Ramon y Cajal, Universidad de Alcala, Instituto Ramon y Cajal de InvestigacionSanitaria (IRYCIS), Madrid, Spain

^36^Teaching Unit of Psychiatry and Psychological Medicine, Department of Medicine, University of Valencia, CIBERSAM, INCLIVA, Valencia, Spain
