# Supplementary material for: Obstetric complications, cortical gyrification, and cognition in first-episode psychosis
Source: Psychol Med. 2025 Jul 22;55:e209. doi: 10.1017/S0033291725100974 (PMC12315652; doi:10.1017/S0033291725100974)
Supplement: Costas-Carrera et al. supplementary material [file S0033291725100974sup001.docx]

Table 2A-Supplementary. Generalized linear model gyrification index lobes by diagnosis (psychosis/control) and stratified by the presence/absence of difficulties during the intrauterine/delivery period (OCs presence/absence) in males.

|  | Diagnosis | | | | Effect  Diagnosis | | Effect  OCs | | Effect  Diagnosis by OCs | | |
| --- | --- | --- | --- | --- | --- | --- | --- | --- | --- | --- | --- |
|  | First Episode Psychosis | | Healthy controls | |  |  |  |  |  |  |  |
|  | OCs - | OCs + | OCs- | OCs+ | X2 Wald | P value | X2 Wald | P value | X2 Wald | P value | FDR adjusted |
|  |  |  |  |  |  |  |  |  |  |  |  |
| Left Frontal LGI | 2.90±0.02 | 2.92±0.02 | 2.92±0.02 | 3.02±0.04 | 4.05 | 0.04* | 5.77 | 0.01* | 3.01 | 0.08^ | 0.20 |
| Right Frontal LGI | 2.91±0.01 | 2.91±0.03 | 2.92±0.02 | 2.99±0.04 | 2.26 | 0.13 | 2.06 | 0.15 | 2.02 | 0.15 | 0.26 |
| Left Parietal LGLI | 3.33±0.02 | 3.31±0.03 | 3.37±0.02 | 3.44±0.05 | 5.55 | 0.01* | 0.72 | 0.39 | 3.00 | 0.08^ | 0.21 |
| Right Parietal LGI | 3.36±0.02 | 3.31±0.03 | 3.38±0.02 | 3.44±0.05 | 3.80 | 0.05^ | 0.12 | 0.73 | 3.10 | 0.08^ | 0.21 |
| Left Temporal LGI | 3.36±0.02 | 3.35±0.03 | 3.41±0.02 | 3.43±0.05 | 2.43 | 0.12 | 0.06 | 0.813 | 0.32 | 0.57 | 0.57 |
| Right Temporal LGI | 3.30±0.02 | 3.28±0.03 | 3.34±0.02 | 3.39±0.05 | 3.94 | 0.05^ | 0.14 | 0.709 | 1.09 | 0.29 | 0.33 |
| Left Occipital LGI | 2.82±0.02 | 2.81±0.03 | 2.86±0.02 | 2.91±0.04 | 4.36 | 0.04* | 0.94 | 0.33 | 1.29 | 0.25 | 0.33 |
| Right Occipital LGI | 2.89±0.02 | 2.87±0.03 | 2.92±0.02 | 2.96±0.04 | 2.47 | 0.12 | 0.17 | 0.68 | 1.11 | 0.29 | 0.33 |
| Left Cingulate LGI | 2.33±0.03 | 2.31±0.03 | 2.36±0.02 | 2.50±0.04 | 11.18 | 0.01* | 5.58 | 0.02* | 8.11 | 0.004* | 0.04* |
| Right Cingulate LGI | 2.36±0.02 | 2.38±0.03 | 2.39±0.02 | 2.49±0.04 | 4.96 | 0.03* | 3.91 | 0.05^ | 2.61 | 0.11 | 0.11 |

FDR: False discovery rate

OCs: obstetric complications +/- (+presence of any stressful event during the intrauterine or delivery period – absence)

LGI: Local gyrification index

Estimated marginal means of predicted gyrification index values are adjusted by covariates, age, sex, and chlorpromazine equivalent mean dose.

Table 2B-Supplementary. Generalized linear model gyrification index lobes by diagnosis (psychosis/control) and stratified by the presence/absence of difficulties during the intrauterine/delivery period (OCs presence/absence) in females.

|  | Diagnosis | | | | Effect  Diagnosis | | Effect  OCs | | Effect  Diagnosis by OCs | | |
| --- | --- | --- | --- | --- | --- | --- | --- | --- | --- | --- | --- |
|  | First Episode Psychosis | | Healthy controls | |  |  |  |  |  |  |  |
|  | OCs - | OCs + | OCs- | OCs+ | X2 Wald | P value | X2 Wald | P value | X2 Wald | P value | FDR adjusted |
|  |  |  |  |  |  |  |  |  |  |  |  |
| Left Frontal LGI | 2.82±0.02 | 2.80±0.04 | 2.87±0.02 | 2.87±0.03 | 3.27 | 0.07^ | 0.26 | 0.61 | 0.05 | 0.83 | 0.97 |
| Right Frontal LGI | 2.83±0.02 | 2.81±0.03 | 2.87±0.02 | 2.85±0.03 | 1.67 | 0.19 | 0.40 | 0.52 | 0.03 | 0.86 | 0.97 |
| Left Parietal LGLI | 3.21±0.02 | 3.20±0.04 | 3.27±0.02 | 3.23±0.04 | 1.82 | 0.17 | 0.79 | 0.37 | 0.16 | 0.68 | 0.97 |
| Right Parietal LGI | 3.23±0.03 | 3.22±0.04 | 3.26±0.02 | 3.26±0.04 | 1.46 | 0.22 | 0.03 | 0.85 | 0.03 | 0.86 | 0.97 |
| Left Temporal LGI | 3.26±0.02 | 3.24±0.04 | 3.29±0.02 | 3.29±0.04 | 1.47 | 0.23 | 0.16 | 0.69 | 0.03 | 0.87 | 0.97 |
| Right Temporal LGI | 3.22±0.02 | 3.17±0.04 | 3.24±0.02 | 3.28±0.04 | 3.03 | 0.08^ | 0.09 | 0.77 | 2.04 | 0.15 | 0.97 |
| Left Occipital LGI | 2.74±0.02 | 2.73±0.04 | 2.79±0.02 | 2.73±0.04 | 0.47 | 0.49 | 1.26 | 0.26 | 0.73 | 0.39 | 0.97 |
| Right Occipital LGI | 2.78±0.02 | 2.77±0.04 | 2.86±0.02 | 2.85±0.04 | 4.86 | 0.02^ | 0.22 | 0.64 | 0.01 | 0.93 | 0.97 |
| Left Cingulate LGI | 2.28±0.02 | 2.26±0.03 | 2.28±0.01 | 2.29±0.03 | 0.31 | 0.57 | 0.00 | 0.98 | 0.25 | 0.61 | 0.97 |
| Right Cingulate LGI | 2.30±0.02 | 2.30±0.04 | 2.34±0.02 | 2.35±0.04 | 1.46 | 0.23 | 0.01 | 0.92 | 0.00 | 0.97 | 0.97 |

FDR: False discovery rate

OCs: obstetric complications +/- (+presence of any stressful event during the intrauterine or delivery period – absence)

LGI: Local gyrification index

Estimated marginal means of predicted gyrification index values are adjusted by covariates, age, sex, and chlorpromazine equivalent mean dose.

**Figure 2 Supplementary.** **Mediation analysis models of obstetric complication mechanisms in healthy controls.**

**Causal model**

Obstetric complications

Working memory

Left Cingulate Gyrification

-7.14 (=0.048*)

1.68 (p=0.10)

|  | Estimate | 95% CI Lower | 95% CI Upper | p-value |
| --- | --- | --- | --- | --- |
| ACME | 1.68 | -0.11 | 4.32 | 0.10 |
| ADE | -7.14 | -15.16 | -0.26 | **0.048*** |
| Total Effect | -5.46 | -13.45 | 1.86 | 0.14 |
| Prop. mediated | -0.31 | -3.73 | 2.48 | 0.24 |

ACME: Average Causal Mediation Effect

ADE: Average Direct Effect

Effect decomposition


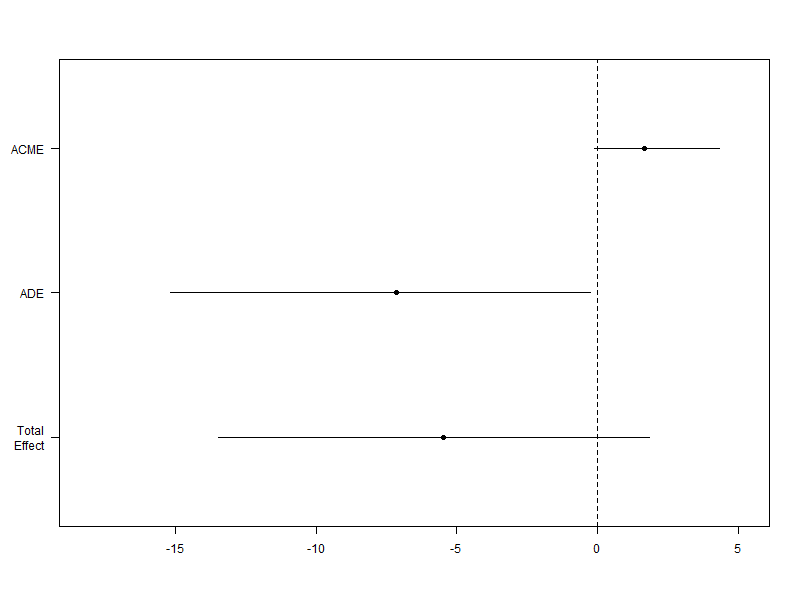


**Figure 3-Suplementary .** **Mediation analysis models of obstetric complication mechanisms in FEP Patients**

**Causal model**

Obstetric complications

Working memory

Left Cingulate Gyrification

-0.48 (p=0.92)

-0.18 (p=0.63)

Parameters

|  | Estimate | 95% CI Lower | 95% CI Upper | p-value |
| --- | --- | --- | --- | --- |
| ACME | -0.18 | -1.32 | 0.41 | 0.63 |
| ADE | -0.48 | -7.11 | 6.77 | 0.92 |
| Total Effect | -0.66 | -7.26 | 6.54 | 0.88 |
| Prop. mediated | 0.27 | -0.91 | 1.35 | 0.88 |

Effect decomposition


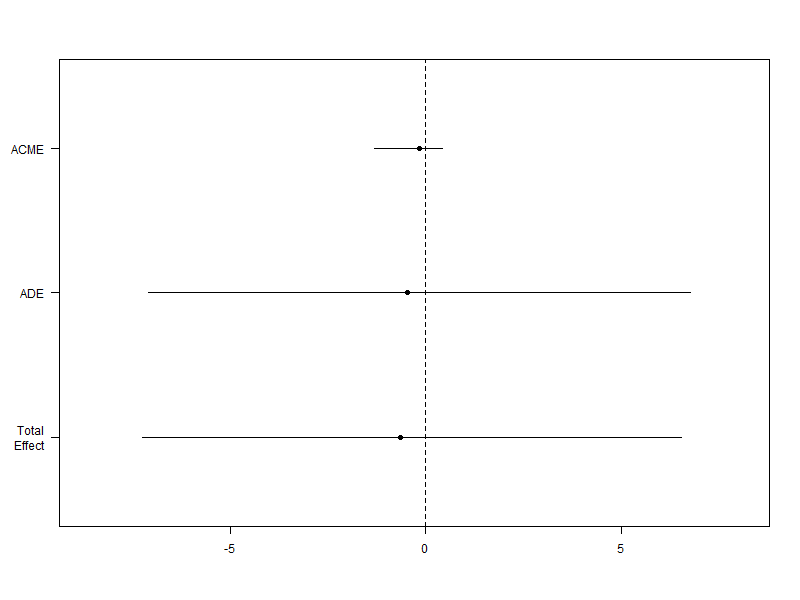


*Causal models of intervention mechanisms, model parameters, and effect decomposition. The causal models panel shows the hypothesized mechanisms of each variable. The effect decomposition panel shows how the average total effect of the variable on the outcome is decomposed into the indirect effect, and the direct effect.

Figure 4: Supplementary Working Memory mediation analysis stratified by sex

Healthy Control male Healthy Control female


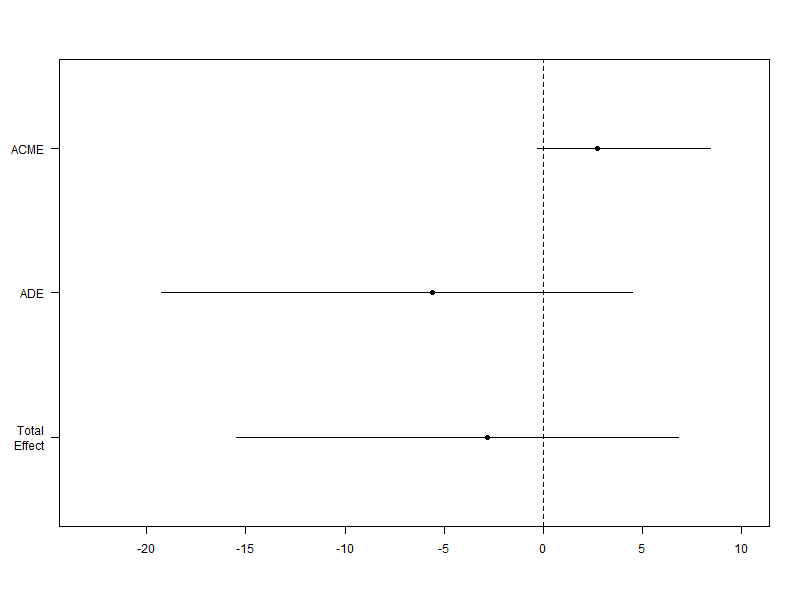

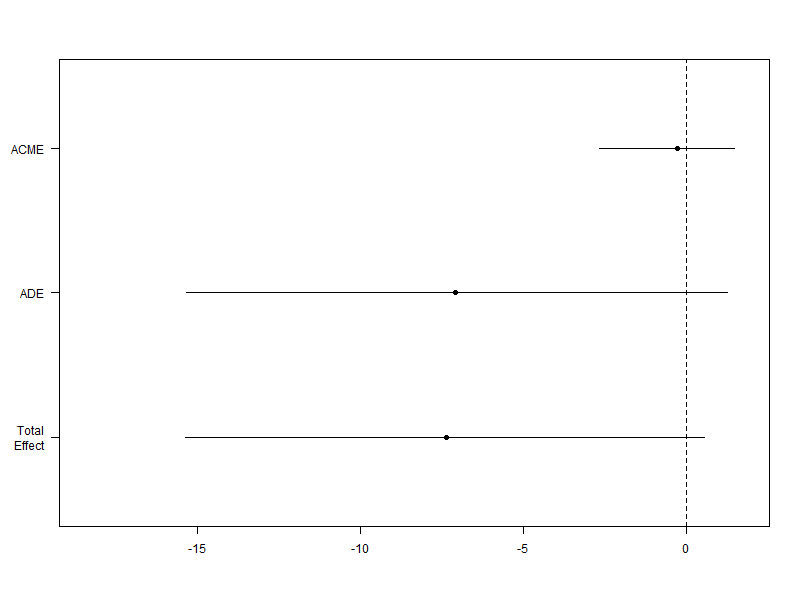


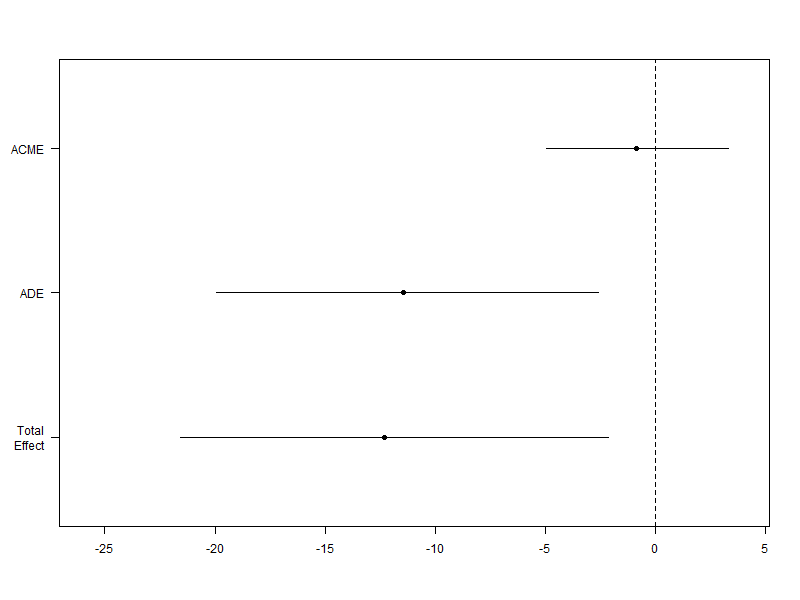

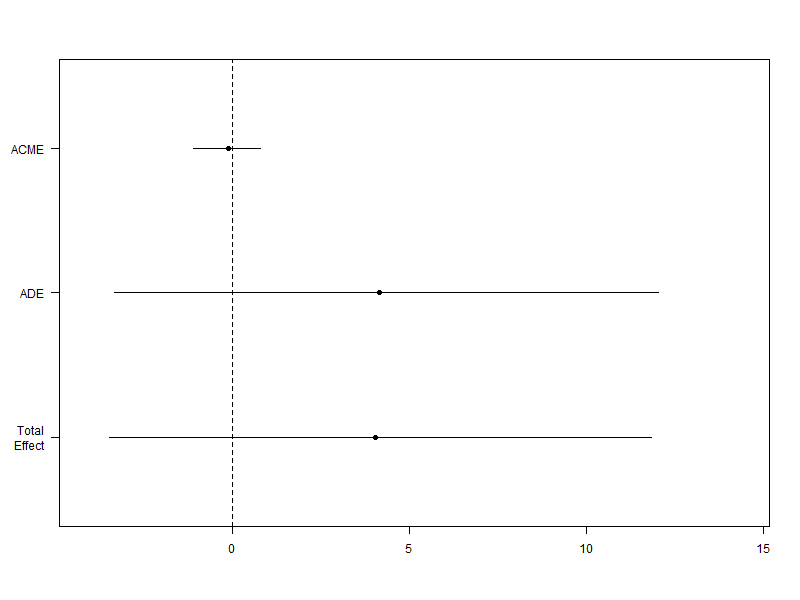
FEP Male FEP Female

Figure 4 Supplementary Working Memory mediation analysis stratified by sex

WM HC Female

|  | Estimate | 95% CI Lower | 95% CI Upper | p-value |
| --- | --- | --- | --- | --- |
| ACME | -0.28 | -2.69 | 1.44 | 0.75 |
| ADE | -7.08 | -15.31 | 1.24 | 0.11 |
| Total Effect | -7.36 | -15.35 | 0.54 | *0.07^ (0.068)* |
| Prop. mediated | 0.04 | -0.40 | 0.95 | 0.77 |

WM HC Male

|  | Estimate | 95% CI Lower | 95% CI Upper | p-value |
| --- | --- | --- | --- | --- |
| ACME | 2.75 | -0.28 | 8.41 | 0.10 |
| ADE | -5.58 | -19.25 | 4.48 | 0.38 |
| Total Effect | -2.83 | -15.49 | 6.84 | 0.66 |
| Prop. mediated | -0.97 | -7.43 | 8.48 | 0.70 |

WM FEP Female

|  | Estimate | 95% CI Lower | 95% CI Upper | p-value |
| --- | --- | --- | --- | --- |
| ACME | -0.85 | -4.95 | 3.32 | 0.62 |
| ADE | -11.45 | -19.92 | -2.57 | **0.02*** |
| Total Effect | -12.30 | -21.57 | -2.15 | **0.03*** |
| Prop. mediated | 0.07 | -0.45 | 0.46 | 0.61 |

WM FEP Male

|  | Estimate | 95% CI Lower | 95% CI Upper | p-value |
| --- | --- | --- | --- | --- |
| ACME | -0.11 | -1.10 | 0.80 | 0.81 |
| ADE | 4.15 | -3.34 | 12.03 | 0.32 |
| Total Effect | 4.04 | -3.47 | 11.85 | 0.32 |
| Prop. mediated | -0.03 | -0.73 | 0.92 | 0.90 |

Figure 5 Supplementary Verbal Memory mediation analysis stratified by sex

VM HC Male VM HC Female


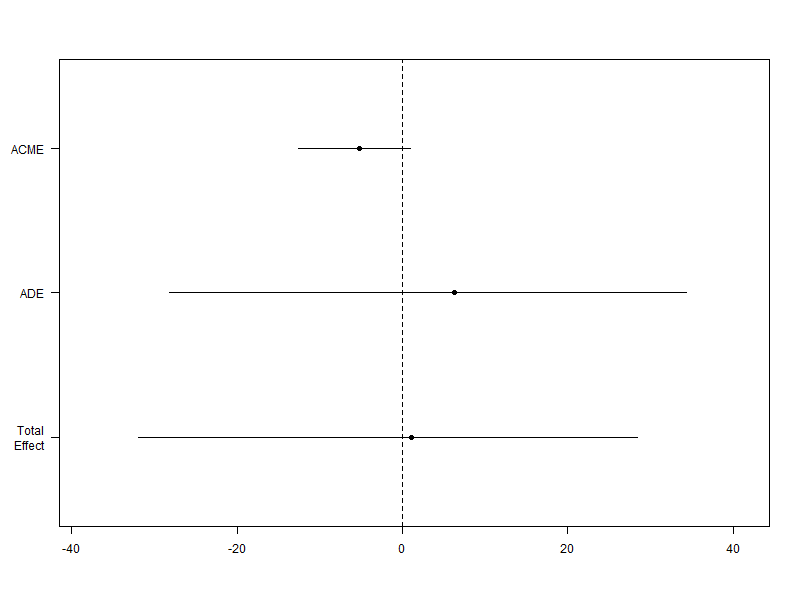

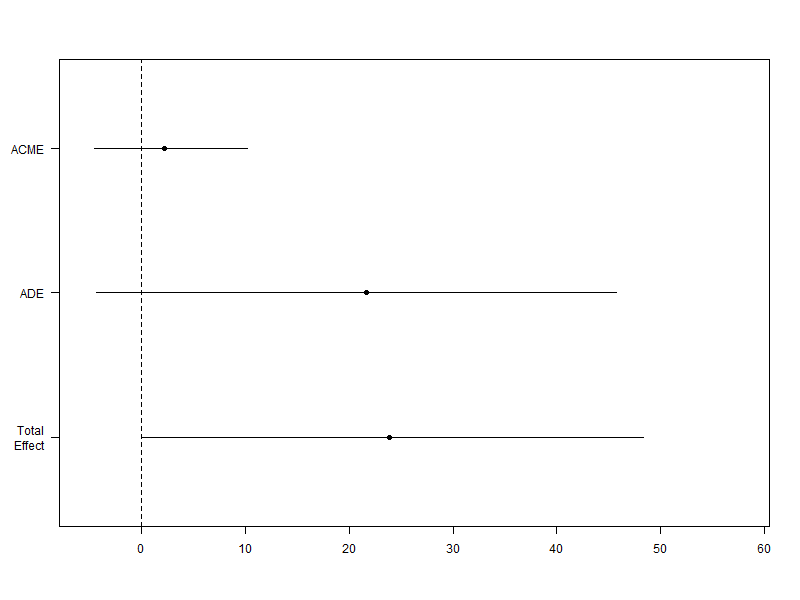


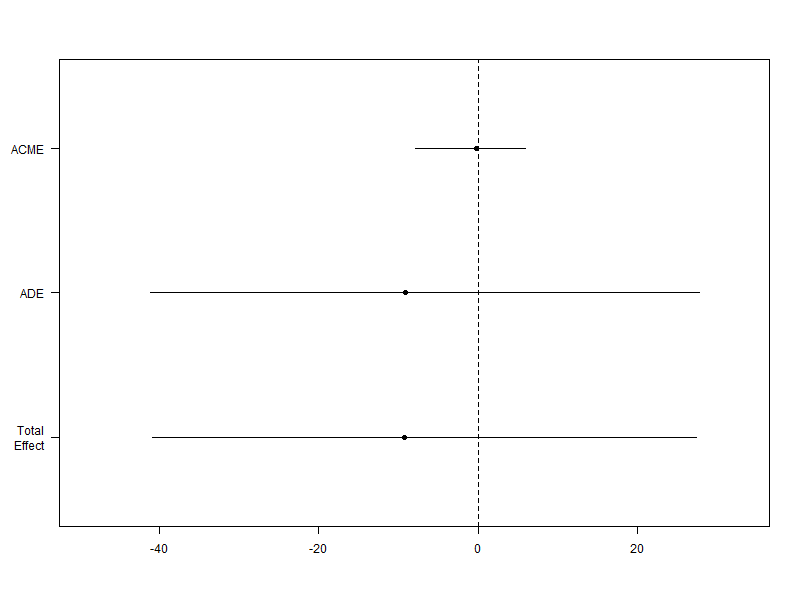

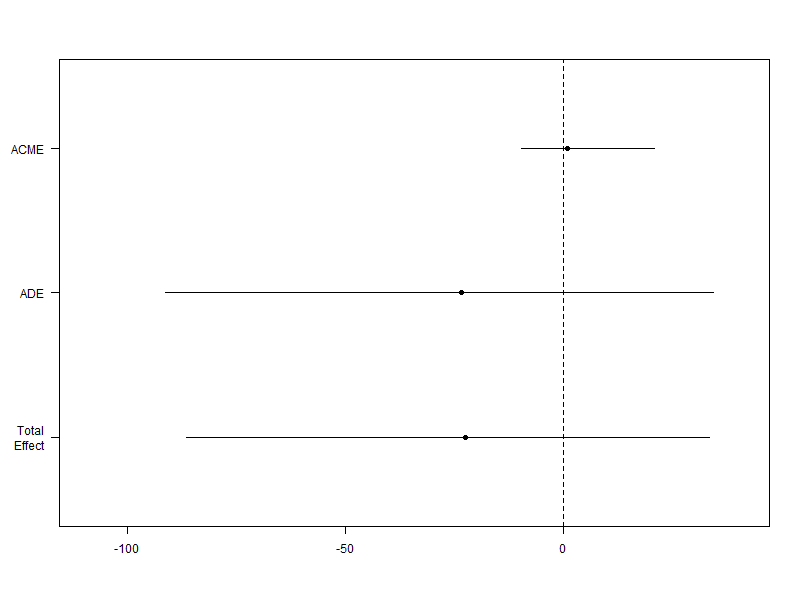
VM FEP Male VM FEP Female

VM HC Female

|  | Estimate | 95% CI Lower | 95% CI Upper | p-value |
| --- | --- | --- | --- | --- |
| ACME | 2.23 | -4.51 | 10.25 | 0.49 |
| ADE | 21.65 | -4.30 | 45.73 | 0.08^ |
| Total Effect | 23.88 | -0.04 | 48.31 | 0.05^ (0.052) |
| Prop. mediated | 0.09 | -0.39 | 0.64 | 0.53 |

VM HC Male

|  | Estimate | 95% CI Lower | 95% CI Upper | p-value |
| --- | --- | --- | --- | --- |
| ACME | -5.18 | -12.54 | 0.94 | 0.11 |
| ADE | 6.25 | -28.21 | 34.37 | 0.69 |
| Total Effect | 1.07 | -31.95 | 28.37 | 0.90 |
| Prop. mediated | -4.82 | -5.61 | 5.50 | 0.90 |

VM FEP Female

|  | Estimate | 95% CI Lower | 95% CI Upper | p-value |
| --- | --- | --- | --- | --- |
| ACME | 0.80 | -9.60 | 20.84 | 0.78 |
| ADE | -23.39 | -91.34 | 34.30 | 0.46 |
| Total Effect | -22.59 | -86.34 | 33.35 | 0.49 |
| Prop. mediated | -0.04 | -2.06 | 1.56 | 0.88 |

VM FEP Male

|  | Estimate | 95% CI Lower | 95% CI Upper | p-value |
| --- | --- | --- | --- | --- |
| ACME | -0.19 | -7.87 | 5.91 | 0.97 |
| ADE | -9.12 | -41.05 | 27.70 | 0.66 |
| Total Effect | -9.31 | -40.89 | 27.39 | 0.63 |
| Prop. mediated | 0.02 | -1.93 | 1.92 | 0.90 |
